# Ambulatory Heart Failure Monitoring: A Systemic Review

**DOI:** 10.7759/cureus.1174

**Published:** 2017-04-18

**Authors:** Muhammad A Mangi, Hiba Rehman, Muhammad Rafique, Michael Illovsky

**Affiliations:** 1 GME Internal Medicine, Orange Park Medical Center; 2 Anesthesiology, Liaquat National Hospital; 3 Cardiology, Orange Park Medical Center

**Keywords:** cardiac implantable electronic device, heart failure hospitalisation, heart failure monitoring

## Abstract

Heart failure (HF) is one of the leading causes of morbidity and mortality and has a large effect on the country’s economy. Although there have been major advances in HF monitoring, including more advanced pharmacological management and device-based therapy, HF-related mortality remains high. It is important to monitor HF so that HF-related hospitalization and mortality can be prevented. Due to the lower sensitivity of clinical features and biochemical markers, as well as the failure of telemonitoring in early detection of HF, more advanced techniques have been sought to more accurately predict impending HF, in order to address timely pharmacological management and prevent heart failure hospitalization (HFH). Device-based therapy has passed through various stages and culminated in the recently introduced CardioMEMS^TM^ (St. Jude Medical, Inc., Saint Paul, Minnesota). CardioMEMS^TM^ is a wireless pulmonary artery pressure (PAP) monitoring device, which continuously monitors PAP and transmits data to a healthcare provider. It rapidly identifies changes in intracardiac pressure and allows timely pharmacological management. CardioMEMS^TM ^showed a higher reduction of HFH compared to any other devices.

## Introduction and background

Heart failure (HF) is a clinical syndrome characterized by the inability of the heart to meet the metabolic demand of the body either by left ventricular dysfunction or right ventricular (RV) dysfunction. The prevalence is 5.8 million in the US and over 23 million worldwide [1]. The incidence is continuously increasing and reaching 650,000 people per year with the financial burden of 30.7 billion dollars [2-3]. Despite the increasing technology of effective pharmacotherapy and device-based therapies for the management of HF, the recurrent hospitalization, morbidity, and mortality remain high [4]. Each admission of acute decompensated HF is the predictor of recurrent admission; i.e., 30% in one month and 50% in six months [1,3]. Most of these admissions are due to the progressive rise in intracardiac filling pressure independent of ejection fraction (EF) and etiology [5-9].

Implantable hemodynamic monitoring device such as CardioMEMS^TM^ (St. Jude Medical, Inc., Saint Paul, Minnesota) detects cardiac filling pressure. These devices detect rising cardiac filling pressure days/weeks before the symptoms, allowing the healthcare provider to intervene in order to prevent HF hospitalizations [10]. Several methods have been tried to reduce heart failure hospitalization (HFH) including weight monitoring, clinical features, biomarkers, and device-based diagnostics, but were not effective in reducing HFH [11-14]. Pulmonary artery pressure (PAP) monitoring has been seen to be effective in reducing HFH in the “CardioMEMS^TM^ Heart Sensor Allows Monitoring of Pressure to Improve Outcomes in New York Heart Association (NYHA) functional Class III Heart Failure Patients” (CHAMPIONS) trial [15]. These effects were persistent in both HF with reduced EF (HFrEF) and HF with preserved EF (HFpEF) [16]. HFH was positively correlated to increasing PAP and decreased with PAP guideline-directed therapy [17]. Hence in 2014, United States Food and Drug Administration (FDA) approved the usage of CardioMEMS^TM^ in the patient with chronic HF, NYHA functional class III to reduce HFH [18].

## Review

### Common methods for HF monitoring

History and Physical

The history and physical examination often help in the diagnosis of HF. The patient often presents with shortness of breath, paroxysmal nocturnal dyspnea, orthopnea, crackles, third heart sound, jugular venous distension, weight gain, and edema, but these features have limited sensitivity and are considered a late manifestation of HF (Table 1). Using symptoms as markers of HF has limited effect on reducing HFH [19]. This management basically involves patient self-assessment and management, including diuretic dose adjustment, which is often an issue in non-compliant and poor self-care skill patients [19]. Telemonitoring has been emerging field in medicine but has failed to show benefit in HFH and mortality [19].

**Table 1:** History and physical examination features for heart failure monitoring

History	Physical
Dyspnea	Jugular venous distension
Paroxysmal nocturnal dyspnea	Hepatojugular reflex positive
Orthopnea	Lung crackles
Cough	S3 heart sound
Weight gain	Edema

Biochemical Markers

Use of biochemical markers to reduced HFH is still under consideration, but data is still under debate. There is no specific threshold level for these markers. Initially, Lainchbury, et al. compared N-­terminal pro-­B-­type natriuretic peptide (NT-­pro-BNP) guided medical therapy with clinically guided medical therapy and usual care, and interestingly found decreased HF-related mortality in patient with NT-pro-BNP guided medical therapy [20]. This benefit was seen in a subgroup of patient ≤75 years old. However, this study did not show any reduction in HFH. McQuade, et al. systemic review data supported the mortality benefit of NT-pro-BNP but also stated that when NT-pro-BNP target is achieved, it decreases HFH [21]. McQuade, et al. data had a specific threshold of NT-pro-BNP (decrease of at least 30%) and BNP (250 pg/ml or less). It was low-strength evidence that stated an association exists between achieving NP predischarge thresholds and reduced HF mortality and readmission. Additionally, Huang, et al. proposed NT-pro-BNP based score which also predicts hospital mortality in HF patients [22]. Another study monitored plasma BNP level in chronic stable HF patient in the ambulatory setting to predict inevitable decompensation and found both asymptomatic and symptomatic HF patient had a wide range of plasma BNP levels [23].

Echocardiography

With further advancement in the medical sciences, echocardiography has been used in the management of HF in order to prevent HFH. It has its own drawback of cost, intra-observer variability, quality of the echocardiographic picture, patient anatomical characteristics, obesity, and accuracy of the test.

Right Heart Catheterization

Right heart catheterization (RHC) has been increasingly used in measuring filling pressure and volume status of the patient, which provide indispensable information in guiding medical therapy in HF patient. However, its utility is limited due to invasive nature and risk of the procedure (Table 2).

**Table 2:** Other methods for heart failure monitoring NT-pro-BNP: N-­terminal pro-­B­-type natriuretic peptide. BNP: B­-type natriuretic peptide.

Other common methods for heart failure monitoring
Telemonitoring
NT­-pro-BNP
BNP
Chest radiograph
Echocardiography
Right heart catheterization

### Cardiac implanted electronic devices for heart failure monitoring

Cardiac implanted electronic devices (CIEDs) are being evaluated to provide ambulatory monitoring of HF. These devices are a closed system consisting of a “sensor” to sense the response and an “effector” to initiate a response. The sensor can take different forms, including a patient, a provider, and/or a device with each form having its own advantage and disadvantage. The main advantage of a device includes continuous monitoring, objective metrics without bias and provides patient specific information (Table 3).

**Table 3:** Cardiac implanted electronic devices for heart failure monitoring SDAAM: Standard deviation of 5-minute median atrial-atrial intervals. SDANN: Standard deviation of 5-minute median ventricular intervals.

Electrophysiological sensors	Hemodynamic sensors
Heart rate variability (SDAAM, SDANN)	Chronicle (right ventricular pressure)
	ePOD (estimated pulmonary artery diastolic pressure)
	Heart PAD (left atrial pressure)
	CardioMEMS^TM^ (pulmonary artery pressure)

Electrophysiological Sensors

Most commonly used CIEDs are pacemaker and defibrillator. They have the capability of sensing certain atrial and ventricular arrhythmias. Through the closed-loop system, they sense and provide appropriate response either pacing or shock. Treating arrhythmias can have a stabilizing effect on HF. Monitoring other electrophysiological variables may have a role in predicting and preventing acute decompensated HF (ADHF). As increased mean heart rate has been demonstrated before ADHF, with a returned back to baseline once ADHF is treated [24]. Such heart rate variability (HRV) can be sensed by the implantable device and therapy initiated to prevent potential hospitalization. HRV can be measured from implantable devices with atrial leads by determining the standard deviation of 5-minute median atrial-atrial intervals (SDAAM) or consecutive ventricular (N-N) intervals (SDANN) over a 24-hour period. These devices detect HRV as early as three weeks before hospitalization [25]. The sensitivity of these devices was 70% in predicting hospitalization.

Hemodynamic Monitoring Sensors

The main pathophysiology of ADHF is a rise in ventricular filling pressure, therefore the main focus of HF management is to lower the ventricular filling pressure without affecting cardiac output. Historically, an RHC was required to monitor hemodynamic status. This led to the development of implantable cardiac filling pressure monitoring devices. These devices have been shown to provide information almost comparable to an RHC [26]. Early work also demonstrated the safety and feasibility of long-term utilization of RV pressure monitoring [27]. More importantly estimated pulmonary artery diastolic (ePAD) pressure monitoring has a stronger correlation in HF as it is a closer surrogate of left atrial filling pressure [28]. Earlier data suggested continuous RV pressure monitoring reduces HFH [29]. Later, Chronicle Offers Management to Patients with Advanced Signs and Symptoms of Heart Failure (COMPASS-HF) trial (Medtronic Inc., Minneapolis, Minnesota) was designed to evaluate therapeutic choices based on RV pressure and its effect on HF hospitalization. However, it did not show any significant effect on hospitalization compared to control group [30]. Secondary analysis of the same study showed 36% relative risk reduction for first HFH. The possible reason of failure to achieve primary endpoint in this study was that the control group might have had a lower than expected number of event. This trial not only helped in reducing HFH but also gave us the insight into the pathophysiology of HF with both reduced and preserved EF. It demonstrated the baseline ventricular filling and ventricular relaxation between groups [10]. In both EF group, patients’ ePAD was significantly higher before the episode of ADHF, but the number of advanced day notice was lower and ventricular filling pressure was significantly higher in patients with preserved EF compared to the patients with low EF. The Reducing Decompensation Events Utilizing Intracardiac Pressures in Patients with Chronic Heart Failure (REDUCE HF trial) combines both devices, implantable hemodynamic monitoring device (IHM) and implantable cardioverter defibrillator to check for RV pressure guided medical therapy [31]. This device also did not show any satisfactory result on reducing HFH. The caveat of this study was, they used IHM device data as a guideline for treatment. Interestingly, continuous measurement of RV pressure is the most straightforward form of implanted hemodynamic monitoring, as we extrapolate pulmonary pressure from RV pressure. However, most patients with ADHF have pulmonary congestion secondary to elevated left atrial pressure (LAP) [32]. Therefore continuous monitoring of LAP may provide a better method of HF monitoring. This discussion continued and in 2010, Hemodynamically Guided Home Self-Therapy in Severe Heart Failure Patients (HOMEOSTASIS) trial demonstrated a reduction of HFH and mortality when treatment was guided on LAP [33]. In this trial, they used implantable Heart POD (St. Jude Medical Inc, Minneapolis, Minnesota). This study formed a basis of a new large population-based ongoing LAPTOP-HF study designed to check the role of implantable left atrial pressure monitoring in association with guiding treatment on HF [34]. This might change the paradigm of HF management.

CardioMEMS^TM ^Monitoring for HF

**Figure 1:**
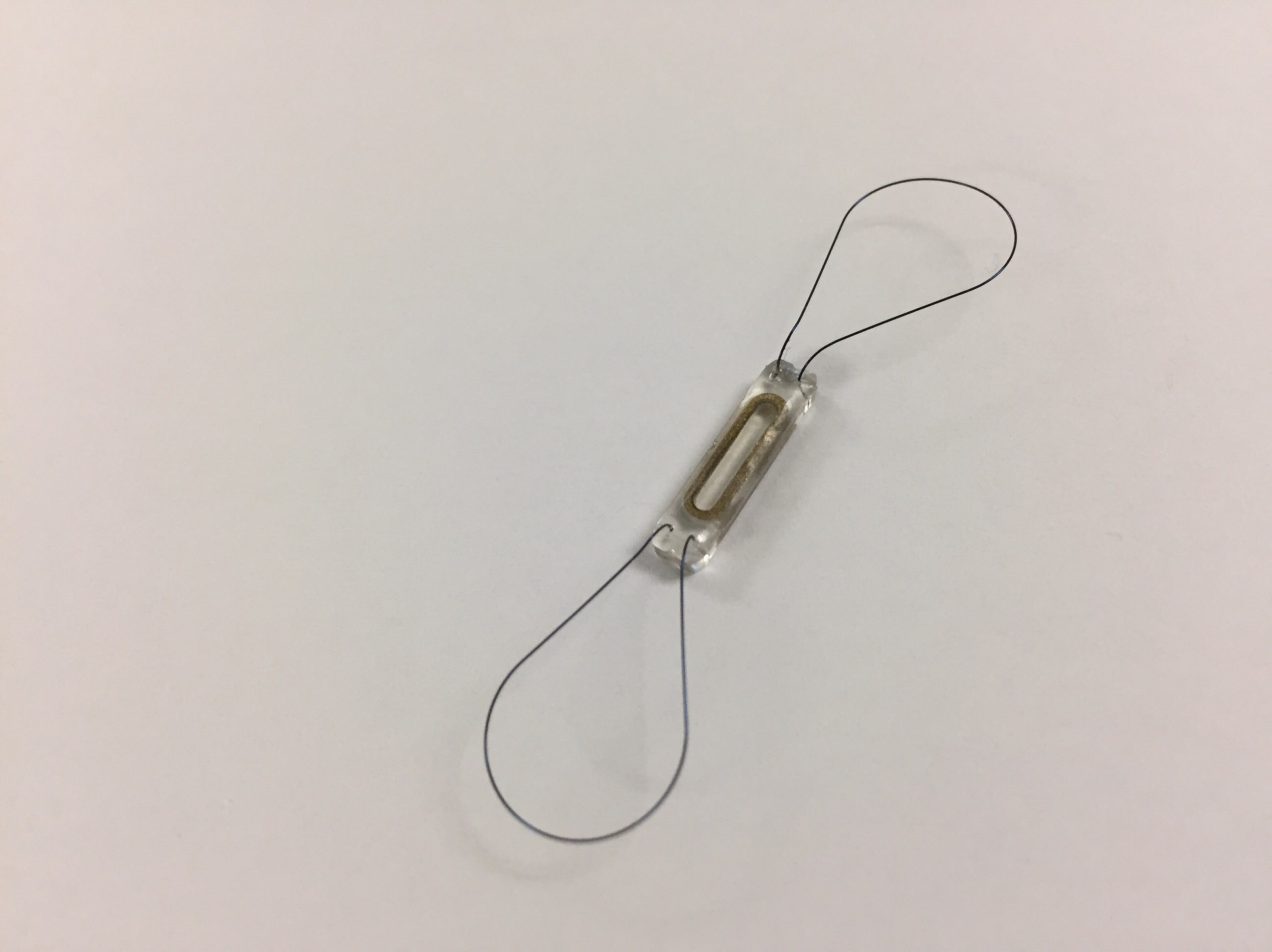
CardioMEMS device

CardioMEMS^TM^ is a wireless device that monitors PAP. It is implanted in the distal pulmonary artery via an RHC. It has been seen that PAP measured by CardioMEMS^TM^ device correlates with PAP measured by Swan-Ganz and echocardiography [35]. The first time CardioMEMS^TM^ was investigated was in the CHAMPIONS trial [36], a multicenter, single-blinded, randomized control trial of 550 patients with NYHA class III HF. The heart failure medications were adjusted on the basis of data generated by the sensor. The CHAMPIONS trial showed a promising result with 28% reduction of HF hospitalization in six months and 37% in 15 months without increasing other causes of hospitalization [37]. The CardioMEMS^TM^ device worked well without any sensor failure and a lower complication rate. This is the first sensor-based device which demonstrated a significantly lower risk of HFH. Due to this success, in 2014 the FDA approved the use of CardioMEMS^TM^ sensor implant in patients with HFpEF and HFrEF with NYHA class III on optimal medical therapy and a history of HFH within the last year [19]. The reduced number of hospitalization in CHAMPIONS trial was related to pressure monitoring via CardioMEMS^TM^ sensor implant which led to appropriate and timely management with dose adjusted diuretics [38]. Subgroup analysis of the study showed the promising result of HF management based on PAP sensor and was more reliable and effective in reducing HFH than HF management base on clinical signs alone [39]. During study follow-up at six months, the patient in the treatment group who had CardioMEMS^TM^ sensor implant received maximal medical therapy including diuretics, vasodilators, and the neurohormonal antagonist [17]. Remote PAP monitoring guided medical therapy reduces hospitalization of the patient with HFrEF with and without cardiac resynchronized therapy (CRT) [40]. This provides additive benefit in CRT patient. There was a significant reduction in all-cause hospitalization and mortality [41]. Surprisingly, measuring PAP with a single RHC alone does not entirely rule out pulmonary hypertension as it underdiagnoses pulmonary hypertension related to left HF [42]. This evidence is further supported by another study in which the author compared HF patient who had implanted hemodynamic device (CardioMEMS^TM^) with no implanted hemodynamic device and reported significant improvement of Kansas city cardiomyopathy questionnaire score and 6-minute walk test in the CardioMEMS^TM^ group [43]. These findings are in favor of the CHAMPIONS trial which has high evidence of reduction of HFH and mortality. Additionally, a recently published retrospective study by Desai, et al. further intensifies the CHAMPIONS trial result [44]. There was a 45% reduction of HFH observed in this study compared to 28% in CHAMPIONS trial. The reduction persisted even in later months. Concomitantly the reduced HFH was not associated with increased risk of all-cause hospitalization (Figure 1).

Improving survival with modern therapy has resulted in HF patients living longer with left ventricular assist devices (LVADs). The author further did sub-analysis of CHAMPIONS trial in order to assess the validity of PAP-directed therapy on optimization of medications, pump parameters, and timing of heart transplantation in the patient receiving LVAD [45]. The data obtained from treatment group revealed more frequent medication adjustment than the control group.

## Conclusions

HF continues to be a major public health problem with a significant financial burden on the country’s economy. As advances in medicine lead to longer life expectancy, more reliable and valid methods will be required to appropriately intervene in order to prevent HF-related hospitalization and death. Implantable hemodynamic devices are the newly emerging tools in the field of HF management. CardioMEMS^TM ^appears to be a leading innovation in the management of HF. However, given a small number of trials with small sample size, larger multicenter trials are required to make valid recommendations in order to reduce HFH, improve the quality of life, and decrease morbidity and mortality related to HF.
